# Research highlight: A critical exploration of the diets of UK disadvantaged communities to inform food systems transformation: a scoping review of qualitative literature using a social practice theory lens

**DOI:** 10.1186/s12889-024-17899-y

**Published:** 2024-03-18

In the UK the food system, encompassing all stages from production to eating, significantly affects societal factors such as social, economic, and environmental aspects. However, this system frequently fails the less fortunate communities, often leaving them with scarce access to affordable, nutritious food. This is a grave concern as unhealthy eating habits are associated with a high number of deaths in Western Europe.

The objective of this study was to evaluate UK-specific qualitative literature studying the diets of less fortunate communities through a social practice theory perspective, a method that aims to deepen the understanding of societal structures and individual actions by examining people’s practices.

This study is a scoping review that reviewed and analyzed literature published in MEDLINE, CINAHL Plus with Full Text, EMBASE, PsycINFO, and Web of Science between January 2010 and May 2021 in the English language. The authors reviewed 45 studies focusing on the diets of individuals residing in less fortunate UK communities.

The study discovered that the diets of these communities were greatly influenced by material factors like transportation, housing, and finances, as well as the local food environment. For instance, limited access to budget-friendly public transportation could restrict access to supermarkets that sell inexpensive food. Additionally, the study found that the meanings and skills linked with food and dietary habits were impacted by mental and physical health issues. Poor mental and physical health could hinder the ability to shop for and cook food.

In conclusion, the research found that the diets of the UK’s less fortunate communities are influenced by a variety of elements, including material conditions, meanings associated with food, and skills related to food preparation and eating.

These results underline the necessity for more comprehensive research and interventions that take into account the broader societal, economic, and environmental factors impacting dietary habits. This could potentially lead to more effective strategies for enhancing the diets of less fortunate communities and promoting fairer food systems.

*The following summaries of hand-selected papers were generated by Springer Nature’s artificial intelligence tool and revised by a subject matter expert to meet Springer Nature's standards.*

